# Chronic Ethanol Exposure Produces Time- and Brain Region-Dependent Changes in Gene Coexpression Networks

**DOI:** 10.1371/journal.pone.0121522

**Published:** 2015-03-24

**Authors:** Elizabeth A. Osterndorff-Kahanek, Howard C. Becker, Marcelo F. Lopez, Sean P. Farris, Gayatri R. Tiwari, Yury O. Nunez, R. Adron Harris, R. Dayne Mayfield

**Affiliations:** 1 Waggoner Center for Alcohol and Addiction Research, The University of Texas at Austin, Austin, Texas, United States of America; 2 Pharmacotherapy Education and Research Center, College of Pharmacy, The University of Texas at Austin, Austin, Texas, United States of America; 3 Charleston Alcohol Research Center, Department of Psychiatry and Behavioral Sciences, Medical University of South Carolina, Charleston, South Carolina, United States of America; CNRS UMR7275, FRANCE

## Abstract

Repeated ethanol exposure and withdrawal in mice increases voluntary drinking and represents an animal model of physical dependence. We examined time- and brain region-dependent changes in gene coexpression networks in amygdala (AMY), nucleus accumbens (NAC), prefrontal cortex (PFC), and liver after four weekly cycles of chronic intermittent ethanol (CIE) vapor exposure in C57BL/6J mice. Microarrays were used to compare gene expression profiles at 0-, 8-, and 120-hours following the last ethanol exposure. Each brain region exhibited a large number of differentially expressed genes (2,000-3,000) at the 0- and 8-hour time points, but fewer changes were detected at the 120-hour time point (400-600). Within each region, there was little gene overlap across time (~20%). All brain regions were significantly enriched with differentially expressed immune-related genes at the 8-hour time point. Weighted gene correlation network analysis identified modules that were highly enriched with differentially expressed genes at the 0- and 8-hour time points with virtually no enrichment at 120 hours. Modules enriched for both ethanol-responsive and cell-specific genes were identified in each brain region. These results indicate that chronic alcohol exposure causes global ‘rewiring‘ of coexpression systems involving glial and immune signaling as well as neuronal genes.

## Introduction

Long-term alcohol use and dependence alter brain function and are linked to persistent changes in gene expression [1–3]. Gene expression profiling in human alcoholics [4–6] and rodent models of binge drinking [7–9] and dependence [10–12] have provided insight into the changes in the brain transcriptional landscape resulting from different drinking paradigms; however, to date it is not clear whether transcriptome changes found in animal models of excessive alcohol consumption are consistent with changes found in human alcoholics. Consilience in gene expression would be a key step toward validating animal models by determining commonalities in molecular plasticity between human and rodent brain.

Genomic approaches have successfully identified alcohol-mediated changes in gene expression in animal models of alcoholism [9,13,14]. These studies suggest that distinct patterns of gene expression underlie specific alcohol-related phenotypes. Animal models of excessive consumption have been developed to investigate different stages of the alcohol abuse cycle that ultimately lead to dependence, including continuous two-bottle choice [15,16], drinking in the dark (DID) [17–19] (a model of binge drinking), and chronic intermittent ethanol (CIE) exposure [12,20] (a model of dependence). In general, studies have focused on transcriptional changes at a single time point following alcohol treatment; thus, it is difficult to determine if the changes in expression patterns are transient or longer lasting. CIE vapor exposure can be used to achieve and maintain high blood ethanol concentrations (180–200 mg/dl) in C57BL/6J mice, and it results in increased self-administration of ethanol [21–23]. Transcriptome profiling immediately following CIE exposure, rather than after subsequent bouts of voluntary drinking, could reveal gene expression and gene network changes associated with induction of ethanol dependence and early withdrawal.

We defined global gene expression profiles in amygdala (AMY), nucleus accumbens (NAC), prefrontal cortex (PFC), and liver of C57BL/6J mice exposed to 4 cycles of intermittent ethanol vapor. Tissue was harvested at 3 time points following the last vapor treatment to assess time-dependent changes in gene expression. We identified time-dependent gene clusters in AMY and NAC that were enriched with astrocytes, microglia, and oligodendrocyte cell types. These sets of genes were primarily associated with inflammatory response function. In contrast, the PFC was enriched with neuronal genes and displayed a greater diversity in directional expression changes, suggesting that the PFC is under greater transcriptional regulatory control than the AMY and NAC.

## Materials and Methods

### Ethics Statement

All procedures were approved by the Medical University of South Carolina Institutional Animal Care and Use Committee and adhered to NIH Guidelines. The Medical University of South Carolina animal facility is accredited by the Association for Assessment and Accreditation of Laboratory Animal Care.

### Animals and Chronic Ethanol Inhalation Procedure

Chronic intermittent ethanol vapor exposure (or air) was delivered in Plexiglas inhalation chambers, as previously described [21,22,24] to drug-naïve C57BL/6J (B6) male mice (8 treated and 8 controls per group). B6 mice were utilized because they show significant escalation of drinking when given access to alcohol after vapor exposure [21–23]. Ethanol treatments were performed in the laboratory of Dr. H.C. Becker (Medical University of South Carolina, Charleston, SC, USA). Briefly, ethanol (95%) was volatilized, mixed with fresh air and delivered to the chambers at a rate of 10 L/min to maintain consistent ethanol concentrations (15–20 mg/L air) in the chamber. Before entry into the chambers for each 16-hour exposure period, mice were administered ethanol (1.6 g/kg; 8% w/v) and the alcohol dehydrogenase inhibitor pyrazole (1 mmol/kg; i.p.) in a volume of 20 ml/kg body weight. Control mice were handled similarly, but they received saline and pyrazole and were exposed to air rather than alcohol vapor. The housing conditions in the inhalation chambers were identical to those in the colony room. Chamber ethanol concentrations were monitored daily using a LifeLoc Breathalyzer and airflow was adjusted to maintain ethanol concentrations within the specified range (180–200 mg/dl). The chamber exposure (16 hr/day) was administered in 4 weekly cycles alternated with 1 week in between in which the mice were left undisturbed (mimicking drinking weeks). Animals were sacrificed at 3 time points: 0-, 8- and 120-hours following the last ethanol vapor or air treatment. Brain and liver samples were snap frozen in liquid nitrogen and stored at −80°C until being shipped (frozen) to the Mayfield/Harris lab at University of Texas at Austin.

### Tissue Harvest and RNA Isolation

Frozen brains were placed in a plastic mold containing Optimal Cutting Temperature compound (OCT) and maintained in a mixture of powdered dry-ice and isopentane. A Microm HM550 cryostat (Thermo Scientific, Ontario, CA) was used for sectioning at a thickness of 300 μm. Micropunches were collected from amygdala (AMY; 1.25 mm; combined basolateral and central nucleus), nucleus accumbens (NAC; 1.25 mm; combined core and shell), and prefrontal cortex (PFC; 2.0 mm). See S1 Fig for tissue punch details. Approximately 100 mg of tissue was obtained from the lower lobes of the liver. Total RNA was isolated according to manufacturer’s instructions using the MagMAX-96 Total RNA Isolation Kit (Ambion, Austin, TX). Total RNAs were quantified on a NanoDrop 1000 spectrophotometer (Thermo Fisher Scientific Inc., Rockford, IL), assessed for quality on an Agilent 2200 TapeStation Instrument (Agilent Technologies, Santa Clara, CA), and amplified/biotin-labeled using the Illumina TotalPrep RNA-96 Amplification kit (Ambion, Austin, TX).

### Microarray Analysis

A web-based tool was used to determine the number of arrays required to detect meaningful statistical changes with a power of 0.8 (http://bioinformatics.mdanderson.org/MicroarraySampleSize/). Aliquots of labeled cRNA were sent to the Yale Center for Genome Analysis (West Haven, CT) where they were hybridized to Illumina MouseRef-8 v2 Expression BeadChips (Illumina, Inc., San Diego, CA) according to manufacturer protocols. Since each BeadChip contains 8 independent arrays, samples were hybridized in a group counter-balanced format to minimize batch effects. Each array was hybridized with material obtained from a single animal; thus, 192 arrays were included in the analysis (16 animals x 4 tissues x 3 time points). Each expression array contains approximately 25,600 transcripts representing over 19,100 unique genes. Transcript abundance was measured by fluorescent intensity after scanning. Microarray data have been submitted to the NCBI Gene Expression Omnibus (GEO) (http://www.ncbi.nlm.nih.gov/geo/) under accession number GSE60676.

### Statistics and Bioinformatics

Unless otherwise noted, the data were analyzed using open source software packages from Bioconductor (http://bioconductor.org) designed for the statistical language R (http://www.r-project.org) and Microsoft Excel. The data were first filtered to include only genes with a detection p-value of ≤0.05 that were present on >80% of the arrays. Data pre-processing included a variance stabilization transformation [25] followed by a quantile normalization step [26] using the Bioconductor package lumi [27]. Expression value outliers were removed using Grubbs’ test [28]. Differential expression analysis for each time point was conducted using empirical Bayes moderated t-statistics from the Bioconductor package limma [29] to compare treated and control mice.

Weighted gene coexpression network analysis (WGCNA) was used to investigate the modular structure of the data at a gene network level. The general framework of WGCNA has been described in detail elsewhere [4,30]. This analysis was conducted as described previously [4] with modifications outlined below. Briefly, we constructed a signed network by calculating Pearson correlations for all pairs of genes (across all time points) and the signed similarity (Sij) matrix derived from Sij = (1+cor(xi,xj))/2, where gene expression profiles xi and xj consist of the expression of genes across multiple microarray samples. Sij was then raised to the power β to represent the connection strength. The goal of this step is to emphasize strong correlations and reduce the emphasis of weak correlations on an exponential scale. We chose a power of β = 8–9 so that the resulting networks exhibited approximate scale-free topology (Soft.R.sq = 0.68–070). All genes were hierarchically clustered based on a dissimilarity measure of topological overlap which measures interconnectedness for a pair of genes [4]. The resulting gene dendrogram was used for module detection using the dynamic tree cut method (minimum module size = 100, cutting height = 0.99). Module preservation scores (z-scores based upon a module membership size of 100) were calculated using the WGCNA package and are shown in S1 Table.

Gene ontology terms were identified using the Database for Annotation, Visualization and Integrated Discovery (DAVID) [31,32], and Ingenuity Pathways Analysis (IPA Ingenuity Systems, www.ingenuity.com) was used to identify overrepresented functional pathways of known gene networks and biological functions. Hypergeometric tests were used to evaluate modules and individual data sets for over-representation of cell type-specific genes. Datasets for neurons, astrocytes, microglia, and oligodendrocytes were obtained from previously published work [33,34]. The dataset used to identify enriched immune-related genes is included in S2 Table and was curated from SA Biosciences (QIAGEN). We then used an effect-size based approach to determine the direction and magnitude of ethanol-induced changes (adjusted p≤0.05) for each coexpression module. Mean t-values were calculated for each module, and this analysis was completed for each time point. Mean t-values were based on unique gene symbols within a module.

The Bioconductor package maSigPro [35] was used to identify clusters of genes showing different patterns of expression as a function of time. The method involves a two-step regression approach. First, a global regression model was used to identify differentially expressed genes, and second, a variable selection strategy was applied to identify differences between groups and time-related expression profiles. Prior to running maSigPro, a set of genes was identified in which gene expression was unchanged over time in control animals (AMY, n = 8098; NAC, n = 8323; PFC, n = 7246; Liver, n = 4157 genes). These genes were then used in the overall maSigPro analysis comparing gene expression in treated versus control animals. For data visualization, hierarchical clustering (4 clusters) was used to identify genes with similar expression patterns.

Historically it has been standard practice to verify a subset of microarray-generated gene expression changes using qRT- PCR. However, we did not include such confirmation in the present study because we have used the Illumina platform (including the particular array used in this study) extensively and ‘‘validated” expression differences with independent qRT- PCR experiments in the past. The level of correspondence between the microarray and RT-PCR results exceeds 80% [4,36,37].

## Results

### Gene Expression Changes

**Fig 1:**
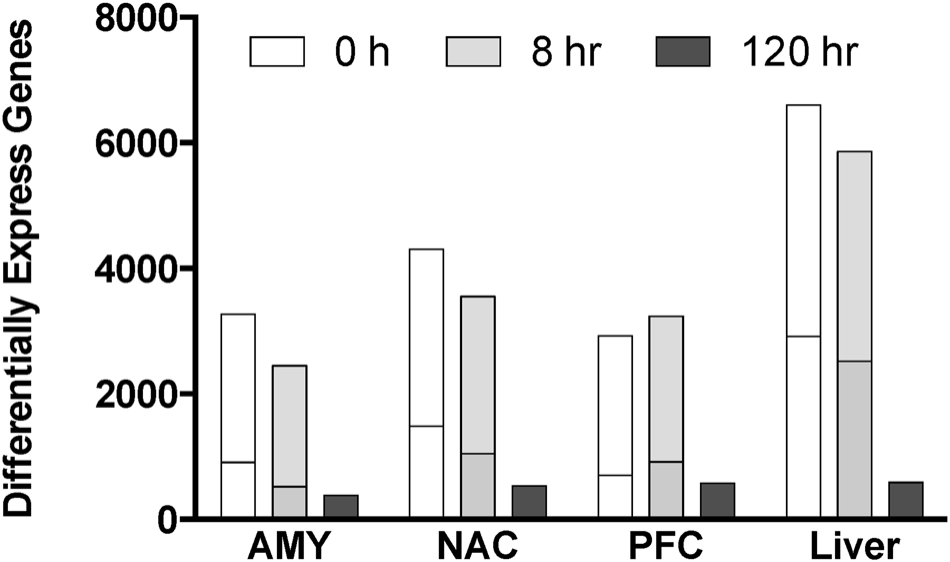
Gene expression changes in brain and liver at three time points following CIE vapor. Bars indicate the number of genes differentially expressed (p≤0.05) in each tissue at 0-, 8-, and 120-hours following CIE vapor treatment. The horizontal lines represent the number of differentially expressed genes after correcting for multiple comparisons (FDR≤0.05).

**Fig 2:**
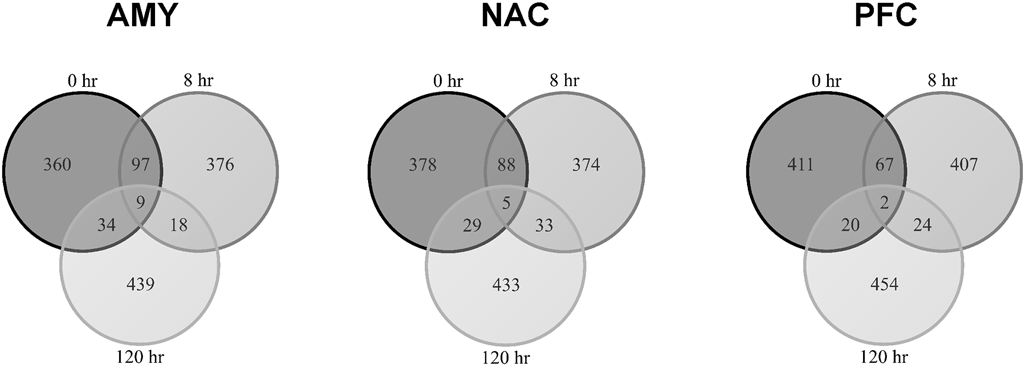
Overlap of the top 500 differentially expressed genes across time. Each time point displays a relatively unique set of differentially expressed genes for each brain region. Times 0 and 8 share the greatest overlap of differentially expressed genes within each brain region; however, this overlap was less than 21%. The three time points combined exhibit very little overlap of differentially expressed genes within each brain region (AMY = amygdala; NAC = nucleus accumbens; PFC = prefrontal cortex).

**Fig 3:**
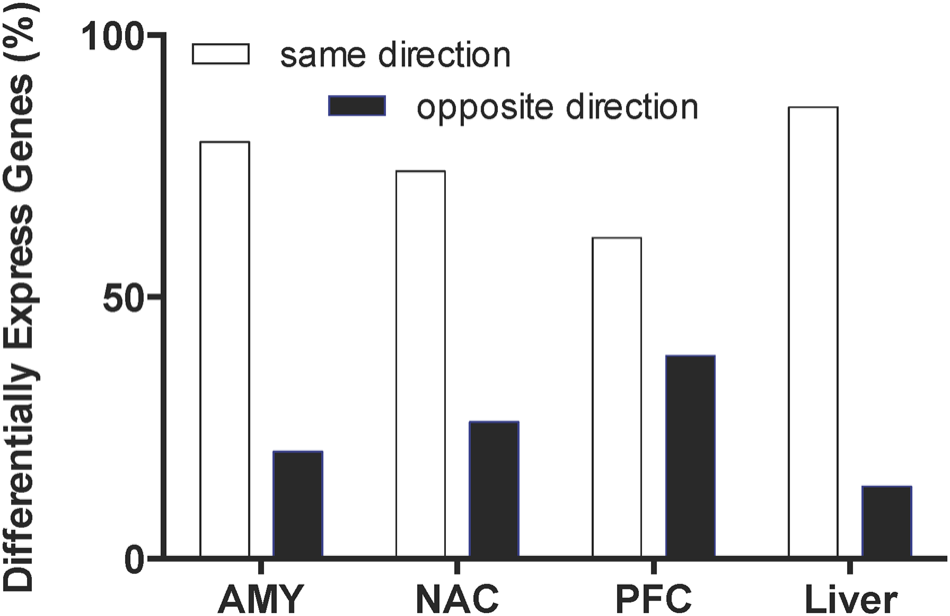
Directionality of differentially expressed genes at 0- and 8-hours. Bars show the percentage of differentially expressed genes (FDR ≤ 0.05) in the same (white) and opposite (black) direction at 0- and 8-hours. Percentages are based on the total number of differentially expressed genes at time 0. (AMY = amygdala; NAC = nucleus accumbens; PFC = prefrontal cortex).

Time- and brain region-dependent changes in gene expression were detected in response to CIE vapor exposure in mice. These procedures do not result in overt behavioral signs of withdrawal at the exposure levels used this study. A similar number of genes were detected in each brain region (9,026–9,185), but fewer genes were detected in liver (7,616). CIE vapor elicited pronounced gene expression changes in all brain regions, as well as in liver (Fig. 1). However, after 120 hours, the transcriptional response to CIE had declined substantially in all tissues when compared with the 0- and 8-hour time points. Differential gene expression was not detected in any of the tissues at the 120-hour time point after multiple comparison correction. The 0- and 8-hour time points showed the greatest overlap of differentially expressed genes across tissues (Fig. 2; S3 Table; note that these data represent the top 500 differentially expressed genes regardless of multiple comparison correction). This overlap was less than 21% for each of the brain regions and about 50% for liver (data not shown). Differentially expressed genes at 0-hour were mostly (60–80%) regulated in a similar direction at 8 hours; however, the consistency of genes changing in the same direction at these time points was greatest for liver and least for PFC (Fig. 3).

### WGCNA and Enrichment Analyses

For all tissues, there was distinct clustering of gene networks at 120-hours compared to other times, and this time point had a greater effect than treatment on sample clustering due to the lack of expression changes (data not shown). WGCNA identified 34–45 modules in brain and 24 modules in liver, with module sizes ranging from 78–1,412 transcripts. The Database for Annotation, Visualization and Integrated Discovery (DAVID) was used for over-representation analysis and to evaluate the biological function of each module.

These results were further substantiated by enrichment analysis using cell-specific and functional gene lists. Published cell-type gene lists (see Methods for details) were used to determine which cells might be disproportionately enriched with differentially expressed genes (adjusted p≤0.05) in response to CIE vapor as a function of time. Enrichment of microglia-specific genes was observed in all brain regions at the 8-hour time point while almost all brain regions and time points were enriched with astrocyte-specific genes (Table 1). The NAC was the only brain region enriched with oligodendrocyte-specific genes, and the PFC was the only region enriched with neuronal genes (Table 1). A list of 824 immune-related genes obtained from the SA Biosciences website (S2 Table) was used to identify over-represented differentially expressed immune genes. For this analysis, an FDR cutoff of 0.05 was used. Notably, there was consistent enrichment of immune-related genes in all brain regions at 8 hours (Table 2). However, there was no over-representation of immune genes in liver (data not shown).

**Table 1:** Enrichment of ethanol-responsive, cell type-specific genes at 0- and 8-hours after CIE vapor.

Tissue	Time Point	Astrocyte	Microglial	Neuron	Oligodendrocyte
**AMY**	0 hr	5.96E-06	7.49E-02	2.17E-01	5.45E-01
	8 hr	7.67E-09	7.14E-07	9.97E-01	9.22E-02
**NAC**	0 hr	4.52E-01	4.52E-01	1.42E-01	4.38E-07
	8 hr	2.62E-03	2.53E-05	4.32E-01	7.42E-03
**PFC**	0 hr	4.80E-04	5.61E-02	4.80E-04	5.95E-01
	8 hr	2.83E-04	1.21E-02	2.39E-02	3.60E-01

Table shows hypergeometric q values for over-representation of cell-type specific genes in differentially expressed genes (adjusted p ≤ 0.05).

AMY = amygdala; NAC = nucleus accumbens; PFC = prefrontal cortex. Q values are shown (hypergeometric test) for differentially expressed genes (FDR≤0.05) that are enriched in specific cell types within each brain region.

**Table 2:** Enrichment of ethanol-responsive, immune-related genes at 0 and 8 hours after CIE vapor.

Tissue	Time Point	P Value
**AMY**	0 hour	2.16E-01
	8 hour	1.23E-05
**NAC**	0 hour	7.05E-01
	8 hour	1.79E-02
**PFC**	0 hour	3.95E-03
	8 hour	1.49E-02

AMY = amygdala; NAC = nucleus accumbens;^c^PFC = prefrontal cortex. P values are shown (hypergeometric test) for differentially expressed immune-related genes (FDR≤0.05) in each brain region.

**Fig 4:**
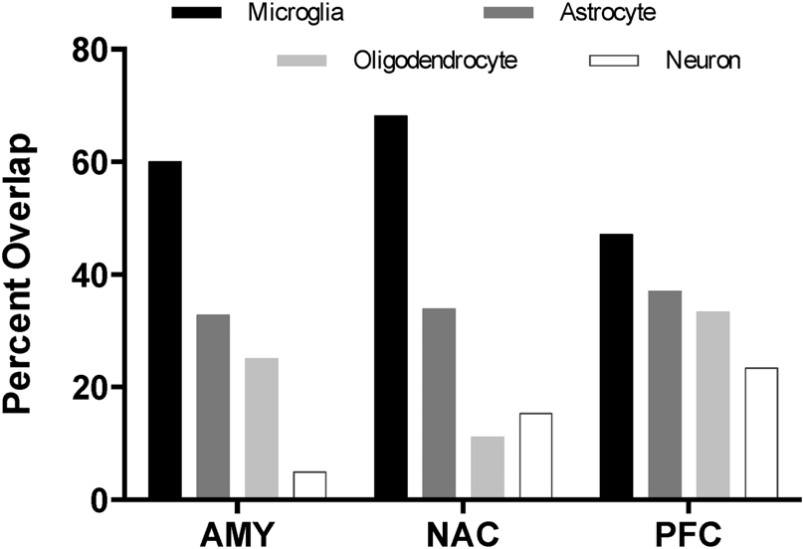
Overlap of cell-specific, differentially expressed genes at 0- and 8-hours after CIE vapor. Bars show the percentage of cell type-specific genes differentially expressed (FDR ≤ 0.05) at both the 0- and 8-hour time points. Percentages are based on the total number of differentially expressed, cell type-specific genes at time 0. (AMY = amygdala; NAC = nucleus accumbens; PFC = prefrontal cortex).

We also examined the overlap of cell-specific differentially expressed genes (FDR≤0.05) at the 0- and 8-hour time points. Microglial genes were the most highly conserved group of cell type-specific genes across these time points for all brain regions (Fig. 4). In addition, modules were evaluated for enrichment with differentially expressed genes. All brain regions exhibited a number of ethanol-responsive modules that were also enriched with genes from one or more of the cell-type/functional gene lists (Table 3). Microglia- and astrocyte-enriched modules showed the most persistent gene expression changes across time and were primarily associated with anti-apoptosis and immune response. In contrast, neuron- and oligodendrocyte-enriched modules showed more transient gene expression changes. For example, neuronal enrichment was only observed in one module (PFC/2) at 0- and 8-hours. A single module (NAC/3) was enriched with microglia and oligodendrocyte genes as well as ethanol-responsive genes at 0- and 8-hours. Table 3 also indicates differentially expressed module hub genes for each brain region and time point. Specific gene names and level of significance is provided in S4 Table.

**Table 3:** Ethanol-responsive WGCNA co-expression modules enriched with cell type-specific genes.

		Astrocytes		Microglia		Neuron		Oligodendrocytes	
*Tissue/ Module*	*Module Function Summary*	*0 hour*	*8 hour*	*0 hour*	*8 hour*	*0 hour*	*8 hour*	*0 hour*	*8 hour*
**AMY/1**	Anti-apoptosis	**X, H** ^**FDR**^	**X, H** ^**FDR**^	**X, H** ^**FDR**^	**X, H** ^**FDR**^				
**AMY/2**	Antigen processing and presentation; adaptive immune response; major histocompatability complex			**X, H**	**X, H** ^**FDR**^				
**AMY/3**	Neuron development; axonogenesis; glycoprotein					**X, H** ^**FDR**^			
**AMY/4**	GPCR activity; nucleotide receptor activity		**X, H** ^**FDR**^						**X, H** ^**FDR**^
**AMY/5**	Glutathione metabolism; drug metabolism		**X, H**						
**NAC/1**	GPCR activity; disulfide bond; immunoglobulin, extracellular matrix	**X, H** ^**FDR**^						**X, H** ^**FDR**^	
**NAC/2**	Glutathione metabolism; drug metabolism	**X, H** ^**FDR**^	**X, H** ^**FDR**^						
**NAC/3**	ras/rho GTPase signaling; anti-apoptosis			**X, H** ^**FDR**^	**X, H** ^**FDR**^			**X, H** ^**FDR**^	**X, H** ^**FDR**^
**NAC/4**	Antigen processing and presentation; adaptive immune response			**X, H** ^**FDR**^	**X, H** ^**FDR**^				
**NAC/5**	Fibronectin, type III subdomain; cell adhesion						**X, H** ^**FDR**^		
**PFC/1**	Antigen processing and presentation; adaptive immune response; major histocompatability complex	**X, H** ^**FDR**^		**X, H** ^**FDR**^					
**PFC/2**	Small GTPase mediated signal transduction; circadian rhythm					**X, H** ^**FDR**^	**X, H** ^**FDR**^		
**PFC/3**	Ribosome; extracellular region; disulfide bond; binding (glycosaminoglycan, pattern, polysaccharide, heparin, carbohydrate); glycoprotein		**X**						

AMY = amygdala; NAC = nucleus accumbens; PFC = prefrontal cortex. Only modules enriched with both cell type-specific and ethanol-responsive genes (FDR ≤ 0.05) are shown. Functional annotations are shown for each module. [An "X" indicates enrichment of the module with ethanol-responsive and cell type-specific genes at the given time point, "H" indicates differentially expressed module Hub gene (unadjusted *p*-value ≤ 0.05), and H^FDR^ indicates differentially expressed Hub gene (FDR ≤ 0.05)].

**Fig 5:**
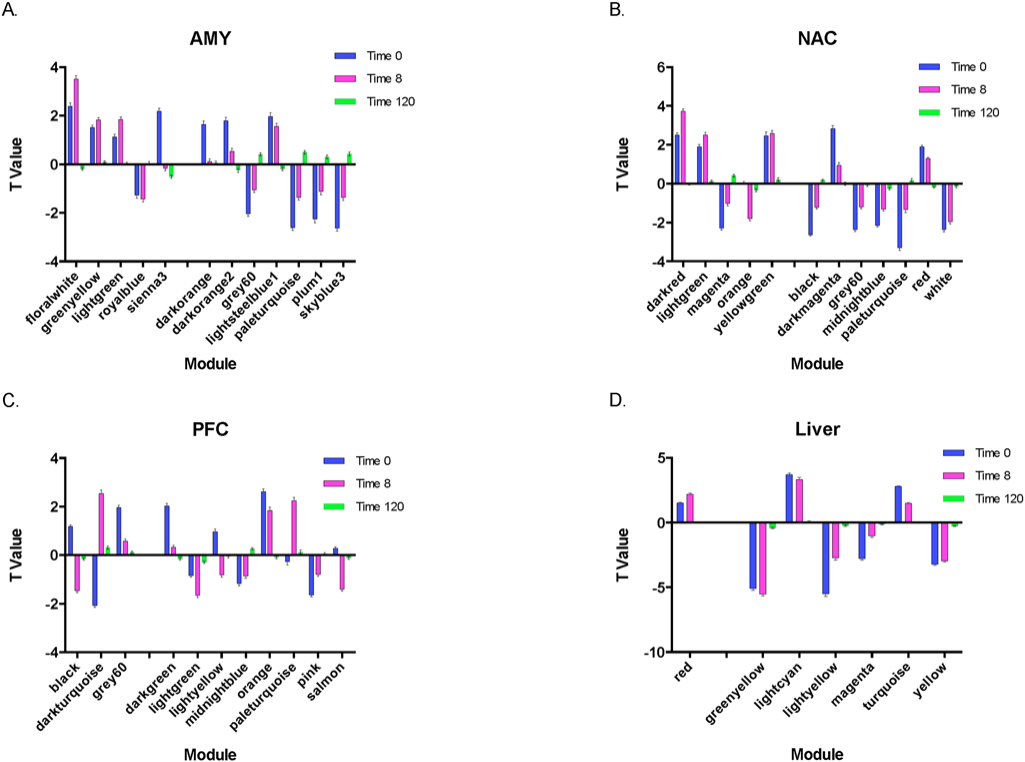
Mean t values for ethanol-responsive modules in brain and liver at 0-, 8-, and 120-hours. Bars show mean t values (±SEM) of ethanol-responsive modules for each tissue at each time point. Panel A = AMY (amygdala), Panel B = NAC (nucleus accumbens), Panel C = PFC (prefrontal cortex), and Panel D = Liver.

To further investigate ethanol-responsive modules, we used an effect-size based approach and determined the direction and magnitude of ethanol-induced changes (adjusted p≤0.05) for each coexpression module. Mean t-values were calculated for ethanol-responsive modules identified by WGCNA at each time point. The magnitude and direction of change were relatively consistent in AMY and NAC at 0- and 8-hours (Fig. 5A and 5B). In contrast, these parameters were quite different in the PFC (black and dark turquoise modules; Fig. 5C). The greatest consistency in t-value magnitude and direction was observed in liver (Fig. 5D).

### Time Series Analysis

**Fig 6:**
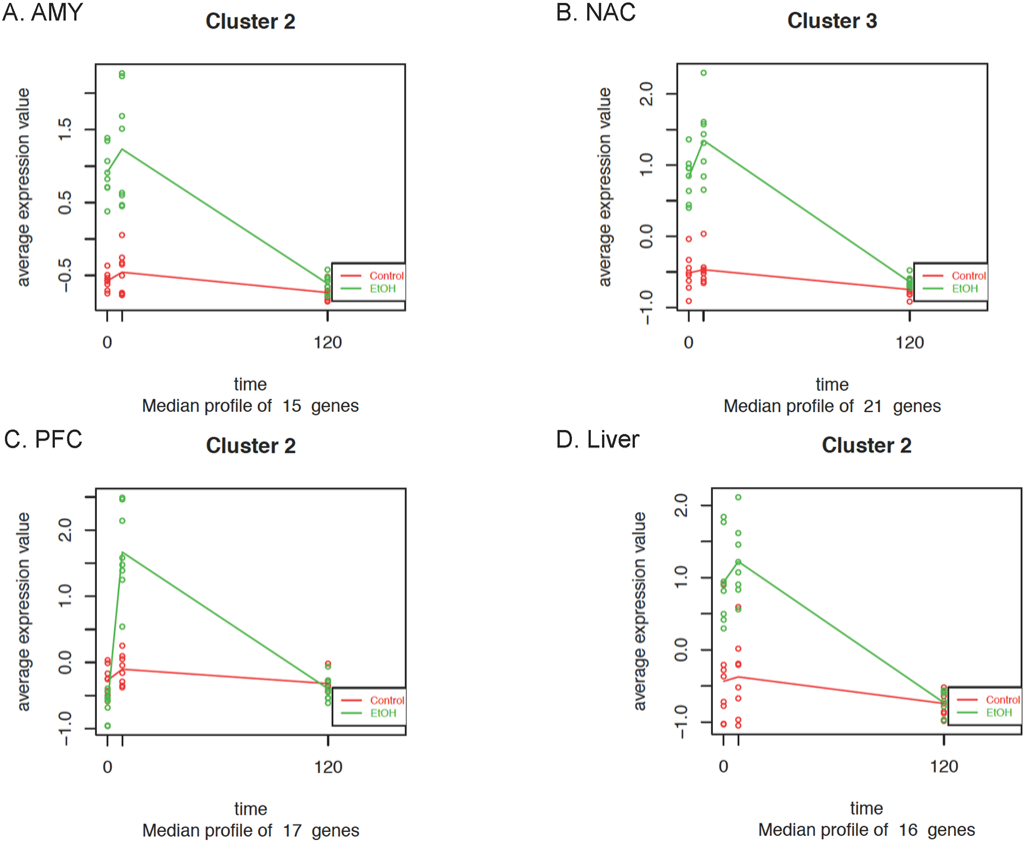
Time series analysis (MaSigPro). A two-step regression approach was used to identify clusters of differentially expressed genes with similar expression patterns across time. Each plot shows the hierarchical clustering (clusters = 4) of average expression profiles by time and tissue type (AMY = amygdala; NAC = nucleus accumbens; PFC = prefrontal cortex; Liver). Panels A-D show gene clusters that have the greatest overlap with WGCNA modules with significantly enriched differentially expressed genes and cell type-specific genes. The dots represent average expression values for each gene in the time series.

A time series analysis was performed using the Bioconductor package maSigPro [35] to identify clusters of differentially expressed genes with similar patterns of expression. For each tissue, the gene clusters with the greatest overlap with WGCNA modules containing significantly enriched differentially expressed and cell type-specific genes are shown in Fig. 6. The complete sets of clusters for each tissue are included in S2–S5 Figs and S5 Table. In the AMY (Cluster 2), all 15 genes are included in WGCNA modules AMY/1 and AMY/2 (Table 3). These modules were significantly enriched with differentially expressed astrocyte and microglia genes (Table 3: AMY/2 and AMY/3). Similarly, in Cluster 3 of NAC, 20 out of 21 genes overlapped with modules enriched with microglia and oligodendrocytes (Table 3; NAC/3 and NAC/4). In contrast, the PFC cluster (Cluster 2) highly overlapped with a module (Table 3; PFC/2) enriched with neuronal genes. The genes included in each maSigPro cluster are shown in Table 4. Additional patterns of differentially expressed genes were detected but were not found to be enriched in WGCNA modules and are not shown (see S2–S5 Figs for the complete set of clusters).

**Table 4:** Clusters of differentially expressed genes with similar expression patterns as a function of time (maSigPro time series analysis).

Tissue	Cluster	Gene Symbol
**AMY**	1	*Hba-a1*, *Asrgl1*, *Hbb-b1*, *Sox21*, *1190002H23Rik*, *Pafah1b3*, *Hist1h4h*, *Mcm5*, *G3bp1*, *Mrp63*, *Trappc4*, *Slc30a10*, *Pcmt1*
	2	B2m*, *Cd74*, *Mfsd2*, *Fcgr2b*, *Psmb9*, *Pglyrp1*, *Htatip2*, *Pcsk4*, *Spp1*, *Sec63*, *EG630499*, *EG667977*, *Alox5ap*, *Ccl25*
	3	*Dtna*, *Slc25a33*
	4	*Rit1*, *Fkrp*, *Fam131a*, *Hes6*
**NAC**	1	*Elac2*, *Dtna*, *Snai3*, *Nrn1l*, *Mpp3*, *Fmo1*, *Hist1h1c*, *Vgf*, *Slc25a3*, *Sorcs3*, *Smap2*
	2	*Hba-a1*, *Heatr1*, *Hbb-b1*, *Sox21*, *AI428936*, *Aldh3b1*, *P2ry1*, *Pex6*, *Phldb1*, *Exdl2*, *H2afj*
	3	H2-T23*, *Mfsd2*, *Scg2*, *Psmb9*, *Pglyrp1*, *Gata2*, *Slc1a2*, *Jun*, *H2-M3*, Htra1*, *Cebpb*, *Il17rc*, *EG630499*, *EG667977*, *Cd151*, *H2-Q7*, *Ccl25*, *Cd74*, *Tsc22d3*
	4	*Slco4a1*, *LOC100046883*, *Sdpr*, *Ppif*
**PFC**	1	*Heatr1*, *Prmt2*, *Tsga14*, *Hbb-b1*, *Trib2*, *4930455F23Rik*, *Dnajb6*
	2	*Bdnf*, *Rem2*, *Adcyap1*, Sorcs3*, *Pglyrp1*, *Fscn1*, Cdkn1a*, *Chst8*, *AI593442*, *Nrn1*, *Ttpal*, *Fam20a*, *Car12*, *Vgf*, *2810452K22Rik*
	3	*LOC381629*, *Arpp21*, *Smpdl3b*, *Tprkb*, *Slc41a3*, *BC028528*, *Ccl25*, *Ppp4r4*, *R74862*, Htra1*, *BC031353*, *Snn*, *EG667977*, *Xpa*, 0610007C21Rik*
	4	*Wwp2*, *Cyhr1*, *Tmem132e*, *C230078M08Rik*, *Lsm10*, *Zmat4*, *Fchsd1*
**Liver**	1	Mrpl48*, *2610528J11Rik*, *Arsb*, *Arsa*, Pex5*, *Dut*, *Dis3l*, *Smad2*, *Ipp*, *Acy3*, *Ndufaf1*, *Lman2*, *Ergic2*, *Adh4*, *Ube2l3*, *Tpk1*, *LOC100048313*, *Atpaf2*, *Snrk*, Pygl*, *Sh3glb2*, *Lrba*, *Tmem93*, *Mrpl22*, *Pnpla7*, *Muted*, *Sec14l2*, *Galt*, Cyb5r3*, *Clec11a*, *F2r*, *Mrps28*, *Mrpl53*, *Rngtt*, *Ppapdc2*, *Sacm1l*, *Trak1*, *Rtn4ip1*, *Zhx1*, *Nelf*
	2	*Akr1b8*, *Vav1*, *Csf1r*, *Sftpd*, *Rcsd1*, *Rims3*, *Lgmn*, *Thy1*, *Clec4f*, *LOC100044439*, *Vcam1*, *Psap*, *Spic*, *Slc16a9*, *Clec4n*, *Btbd11*
	3	*Ubl3*, *Soat2*, *Eps15*, *Rap1b*, *Tbk1*, *Adrb2*, *Epn2*, *Slc2a1*, *Rps9*, *Usp3*, *Nrbp2*, *Cyp2b10*, Cyp3a13*, *Acat1*, *Nr1i2*, *LOC100048445*, *Oas2*, *Gdpd1*, *Cd68*, *Eif3k*, *Sult1a1*, *Rnf166*, *Hgsnat*, *Cyp1a2*, *Tm4sf4*, *Nr1i3*, *Cyb5*, *Cyp2b23*, *Keap1*, *Samd4*, *Nrap*
	4	*9630015D15Rik*, Mgll**, Smarce1*, *Cpt2*

AMY = amygdala; NAC = nucleus accumbens; PFC = prefrontal cortex. Red text indicates clusters of genes overlapping with WGCNA modules that were enriched with differentially expressed and cell type-specific genes. Illumina Probe_Ids resulting in duplicate and triplicate Gene Symbols within the clusters are indicated by * and **, respectively.

## Discussion

The goal of the current study was to determine time-dependent transcriptional changes in brain and liver that result from administration of repeated ethanol vapor. This paradigm has been shown to escalate voluntary drinking in both rats and mice and represents a rodent model of dependence [38]. Our hypothesis was that time- and brain region-dependent changes in expression would be observed, and that at least a subset of genes, would be persistently changed in response to CIE vapor. Animals were sacrificed at 0-, 8-, and 120-hours after the last ethanol treatment to determine whether persistent changes in gene expression would be observed during protracted abstinence. At the 8-hour time point, mice are in acute withdrawal based on hypothalamic-pituitary-adrenocortical axis elevation [39], but they do not display seizures because they are a relatively seizure-resistant strain.

A multi-level analysis approach was utilized which included differential expression, network analysis, cell-type specificity, and time-series clustering analyses. These approaches include computational algorithms that enhance the analysis of gene coexpression networks existing in diverse expression datasets. In addition, since the brain transcriptome is organized into gene modules associated with major cell classes and specific synaptic and cellular functions [34], we classified sets of genes based upon subcellular localization. Neurons and glial cells are characterized by unique transcriptional signatures [33,40] and these signatures can be identified reliably from analysis of complex brain tissue without isolating homogeneous populations of cells [4,34]. Classifying significantly regulated genes into cell type-specific signatures improves the quality of inference and potentially leads to refined hypotheses [41].

A time-series analysis (Bioconductor package maSigPro) was performed to identify clusters of genes with similar expression patterns. Each brain region displayed distinct clusters overlapping with WGCNA modules enriched in differentially expressed and cell type-specific genes. In the AMY, a cluster was identified (Cluster 2: Fig. 6 and Table 4) in which all genes were included with modules enriched with differentially expressed, and enriched with astrocyte and microglia genes. The expression of this cluster of genes was significantly increased at 0- and 8-hours, but returned to baseline at 120-hours. IPA analysis indicated that this cluster was enriched in "Inflammatory Disease"-related genes (*Alox5ap*, *B2m*, *Cd74*, *Fcgr2b*, *Hla-A*, *Pglyrp1*, *Psmb9*, *Spp1*). Interestingly, *B2m* is known to be important for immune responses and has been shown to be alcohol responsive in multiple studies [13,42–44]. Blednov *et al*. [42] demonstrated that deletion *of B2m* reduced ethanol consumption in the limited access two-bottle choice test for ethanol intake, supporting a hypothesis that genes within this cluster may play a role in mediating voluntary drinking. In addition, the innate immune cytokine *Cd74* was a member of this cluster. The expression of this gene is rapidly induced by alcohol and has been linked to the progression of cytokine responses during alcohol withdrawal [45] which is consistent with changes observed 8 hours post-treatment.

In NAC, a cluster of genes overlapped with modules enriched with microglia and oligodendrocytes (Cluster 3: Fig. 6 and Table 4). Similar to the AMY, IPA analysis indicated that this cluster was enriched in "Inflammatory Disease"-related genes (*Cd74*, *Cebpb*, *Hla-A*, *Hla-E*, *Hla-G*, *Htra1*, *Il17rc*, *Pglyrp1*, *Psmb9*, *Slc1a2*). *Slc1a2* (a glial glutamate transporter) is highly expressed in microglia [46] and has been linked to neurodegenerative disorders [47] as well as drug dependence [48]. This gene is differentially expressed in human alcoholic post-mortem brain [4] and drugs acting on this target alter motivation to drink in dependent animals [49]. In addition, genetic variation in glutamate transporters confers risk-taking behavior in alcoholics [50]. Together, these findings support a role for *Slc1a2* in alcohol intake and dependence. As in the AMY, the cytokine *Cd74* was also identified in NAC, suggesting that it may have a role in innate immune responses in multiple brain regions. Other genes in this cluster that are differentially expressed in mouse models and human alcoholics include *Htra1* [13] and *Il17rc* [4]. *Tsc22d3* and *Gata-2* are alcohol-responsive members of this gene set but are not members of inflammatory response pathways. *Tsc22d3* functions as a transcriptional regulator and is differentially expressed in human alcoholics [4]. *Tsc22d3* may be associated with neuroplastic changes in response to drugs of abuse, including ethanol in mouse striatum [51].

In contrast to the glial signature of many of the clusters, one PFC gene cluster (Cluster 2: Fig. 6 and Table 4) was highly overlapping with a WGCNA module enriched with differentially expressed neuronal genes. Mean t-value distributions (Fig. 5) indicated that the magnitude and direction of change were clearly different in the PFC compared to the other brain regions, showing both a significant down- and up-regulation of genes at the 0- and 8-hour time points. These data suggest that chronic ethanol vs withdrawal changes in neuronal gene regulation may reflect greater transcriptional control in PFC than in the enriched cell types in AMY and NAC. The most prominent pathway identified by IPA analysis indicated that this cluster was enriched in "Neurological Disease"-related genes (*Adcyap1*, *Bdnf*, *CA12*, *Cdkn1a*, *Vgf*). *Bdnf* has a well-documented role in synaptic plasticity [52,53] and addiction [54]. We recently demonstrated that *Bdnf* is significantly down-regulated in homogenized medial PFC tissue [11] as well as in purified synaptoneurosome preparations [55]. Reductions of *Bdnf* levels [56,57] and knockdown of *Bdnf* expression increase ethanol-drinking behavior [58]. Together, these data support a role for *Bdnf* in the modulation of ethanol intake. Alcohol consumption is known to be modulated by circadian-related cellular function [59]. In the current study, the circadian gene *Adcyap1* was identified as differentially expressed member of an enriched neuronal module and may represent a regulatory mechanism involved in the time-dependent expression changes.

These marked changes in glial, and particularly microglial, genes at both the 0- and 8-hour time points support an emerging concept of neuroimmune changes during alcohol dependence [60,61]. Alterations in neuroimmune gene expression are seen in human alcoholics and also in rodent models with intermittent administration of high doses of ethanol [5,62,63]. It is interesting to note that chronic voluntary alcohol consumption (resulting in lower blood levels compared to inhalation or injection of ethanol) did not produce pronounced changes in expression of microglial or neuroimmune genes in a tissue homogenate [9], but such changes were more prevalent in a synaptoneurosome preparation that enriches for synaptic elements, including glia [55]. It will be interesting to examine in future studies whether voluntary alcohol consumption in this CIE model influences the gene expression profile reported in the present study.

The present study shows marked changes in gene expression between the 0- and 8-hour time points in brain neuronal compartments, but fewer differences in non-neuronal clusters, and very few in liver. This is consistent with marked changes in neuronal excitability during the first 8 hours following withdrawal of alcohol and emphasizes the importance of comparing neuronal and glial gene expression patterns, as the glial changes are much more stable during withdrawal than the neuronal clusters.

In conclusion, gene modules and time-dependent gene clusters were identified in AMY and NAC that were enriched with astrocytes, microglia, and oligodendrocyte cell types. These sets of genes were primarily associated with inflammatory response function and the changes in expression in this model of alcohol dependence is consistent with the proposed role of neuroimmune signaling in promoting alcohol consumption [42,60]. In contrast, the PFC was enriched with neuronal genes and displayed a greater diversity in directional expression changes, suggesting that PFC is under greater transcriptional regulatory control. Importantly, of the many expression changes identified at 0- and 8-hours after ethanol vapor treatment, the majority of changes returned to baseline levels after 120-hours. These results suggest that although intermittent ethanol vapor treatment increases alcohol consumption 120 hours after the last treatment, it is difficult to detect changes in gene expression that might be responsible for signs of protracted abstinence. It is possible that some of the weak changes observed in our study after 120 hours and by Tapocik *et al*. [10] after 3 weeks of abstinence are important but are diluted by the cellular heterogeneity of the brain. Analysis of cell-specific transcriptomes, or use of preparations such as synaptoneurosomes [55], may be needed to detect persistent changes in gene expression produced by alcohol dependence.

## Supporting Information

S1 Fig Tissue Micropunches. Frozen brains were placed in a plastic mold containing OCT and maintained in a mixture of powdered dry-ice and isopentane. A Microm HM550 cryostat (Thermo Scientific, Ontario, CA) was used for sectioning at a thickness of 300 μm. Micropunches were collected from amygdala (AMY; 1.25 mm; combined basolateral and central nucleus), nucleus accumbens (NAC; 1.25 mm; combined core and shell), and prefrontal cortex (PFC; 2.0 mm).
(PDF)

S2 Fig Time series analysis (MaSigPro). A two-step regression approach was used to identify clusters of differentially expressed genes with similar expression patterns across time. Each plot shows the hierarchical clustering (clusters = 4) of average expression profiles by time in the amygdala (AMY). The dots represent average expression values for each gene in the time series.
(PDF)

S3 Fig Time series analysis (MaSigPro). A two-step regression approach was used to identify clusters of differentially expressed genes with similar expression patterns across time. Each plot shows the hierarchical clustering (clusters = 4) of average expression profiles by time in the nucleus accumbens (NAC). The dots represent average expression values for each gene in the time series.
(PDF)

S4 Fig Time series analysis (MaSigPro). A two-step regression approach was used to identify clusters of differentially expressed genes with similar expression patterns across time. Each plot shows the hierarchical clustering (clusters = 4) of average expression profiles by time in the prefrontal cortex (PFC). The dots represent average expression values for each gene in the time series.
(PDF)

S5 Fig Time series analysis (MaSigPro). A two-step regression approach was used to identify clusters of differentially expressed genes with similar expression patterns across time. Each plot shows the hierarchical clustering (clusters = 4) of average expression profiles by time in the Liver. The dots represent average expression values for each gene in the time series.
(PDF)

S1 Table WGCNA module preservation scores (z-scores based upon a module membership size of 100). (XLSX)

S2 Table Immune-responsive gene list used in enrichment analysis. (XLSX)

S3 Table Top 500 changed genes in AMY, NAC, and PFC. (XLSX)

S4 Table Differentially expressed WGCNA module hub genes. (XLSX)

S5 Table Gene information for maSigPro clusters shown in Fig. 6. (XLSX)
