# Supplementary material for: Chronic Ethanol Exposure Produces Time- and Brain Region-Dependent Changes in Gene Coexpression Networks
Source: PLoS One. 2015 Mar 24;10(3):e0121522. doi: 10.1371/journal.pone.0121522 (PMC4372440; doi:10.1371/journal.pone.0121522)
Supplement: S1 Fig — Frozen brains were placed in a plastic mold containing OCT and maintained in a mixture of powdered dry-ice and isopentane. A Microm HM550 cryostat (Thermo Scientific, Ontario, CA) was used for sectioning at a thickness of 300 μm. Micropunches were collected from amygdala (AMY; 1.25 mm; combined basolateral and central nucleus), nucleus accumbens (NAC; 1.25 mm; combined core and shell), and prefrontal cortex (PFC; 2.0 mm). (PDF) [file pone.0121522.s001.pdf]

Micropunch Coordinates: PFC

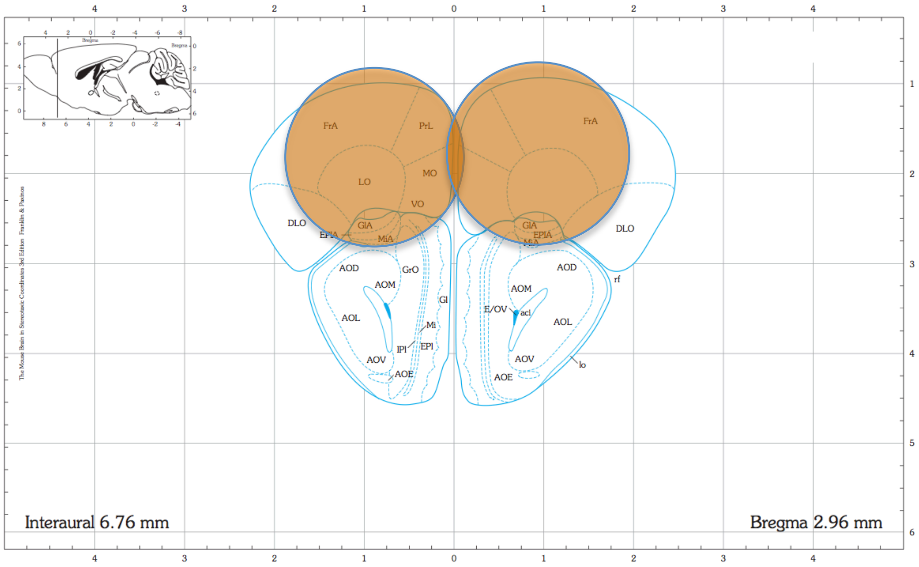

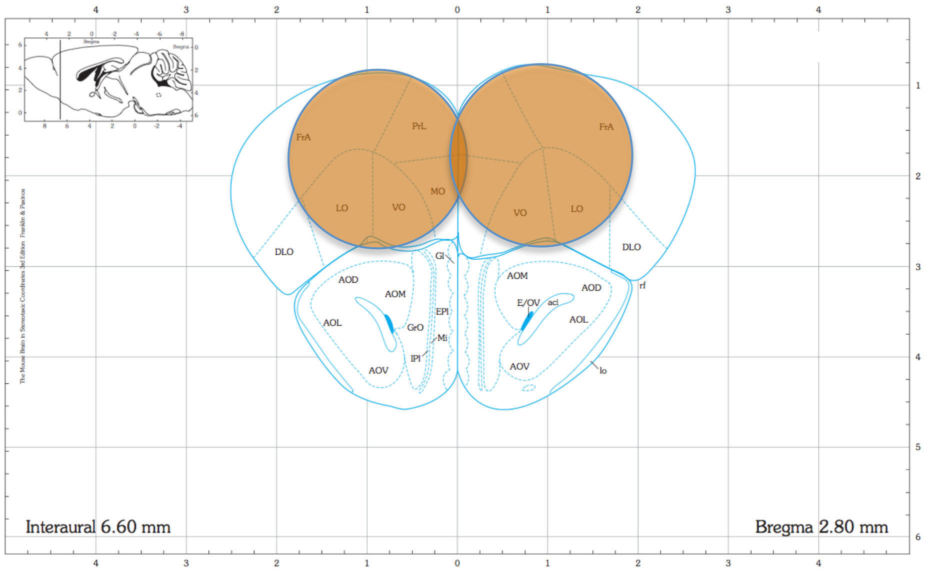

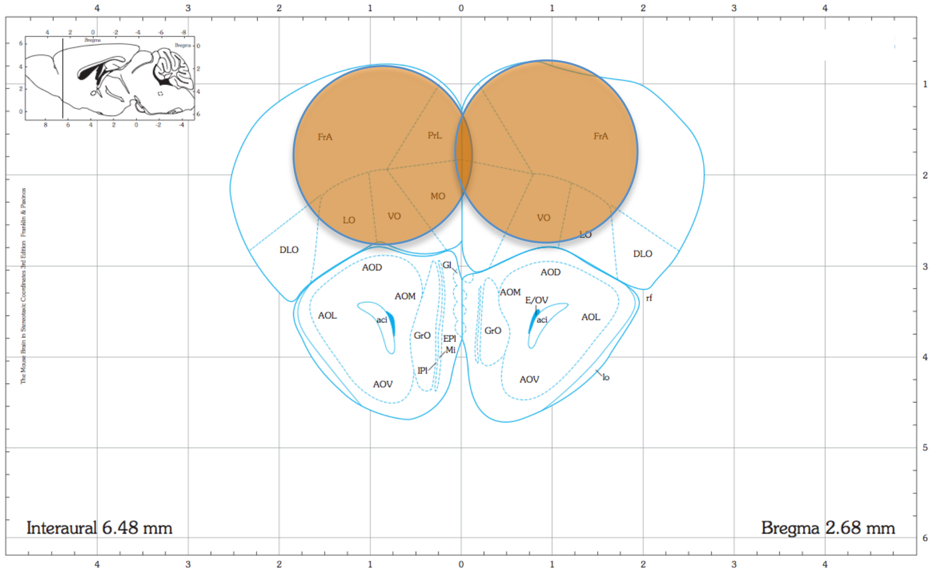

Bregma (+3.00)..(+2.70)

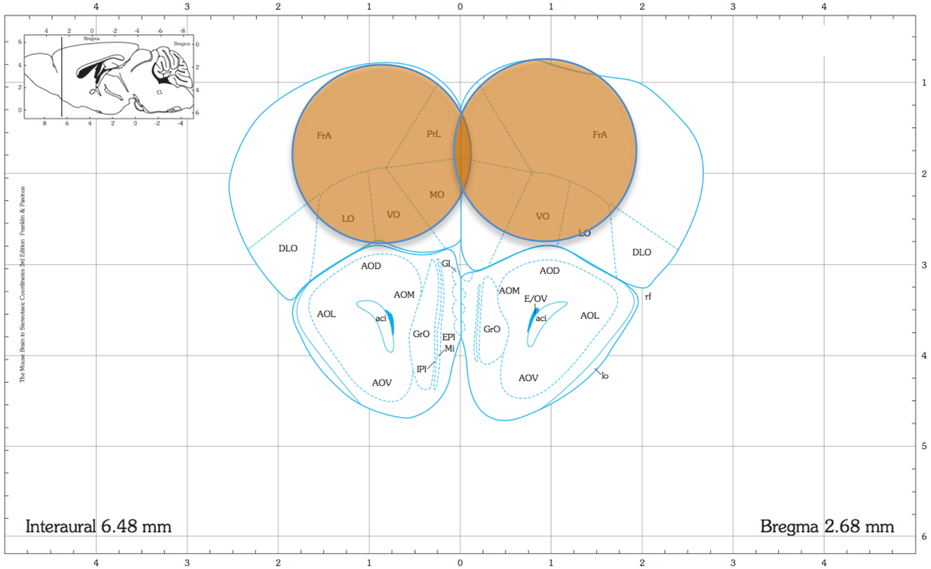

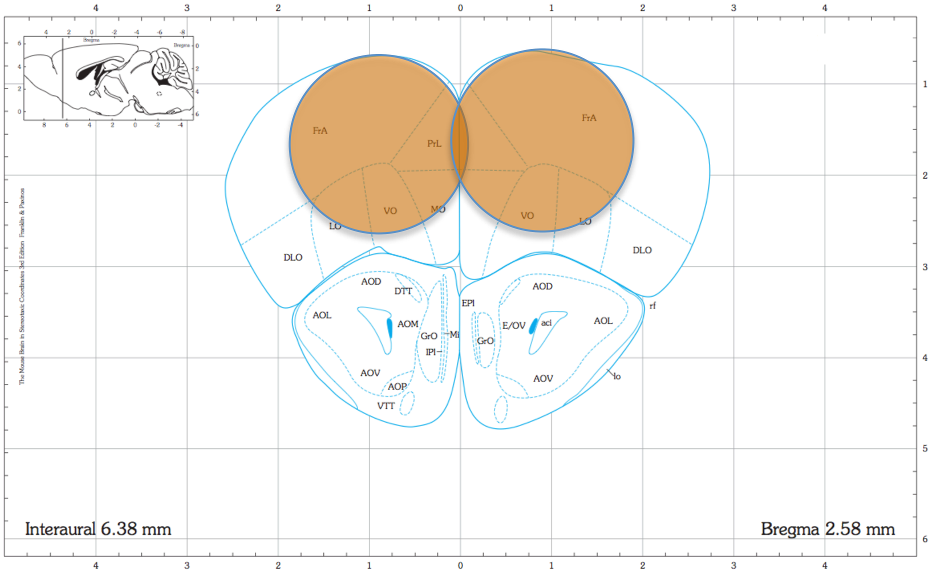

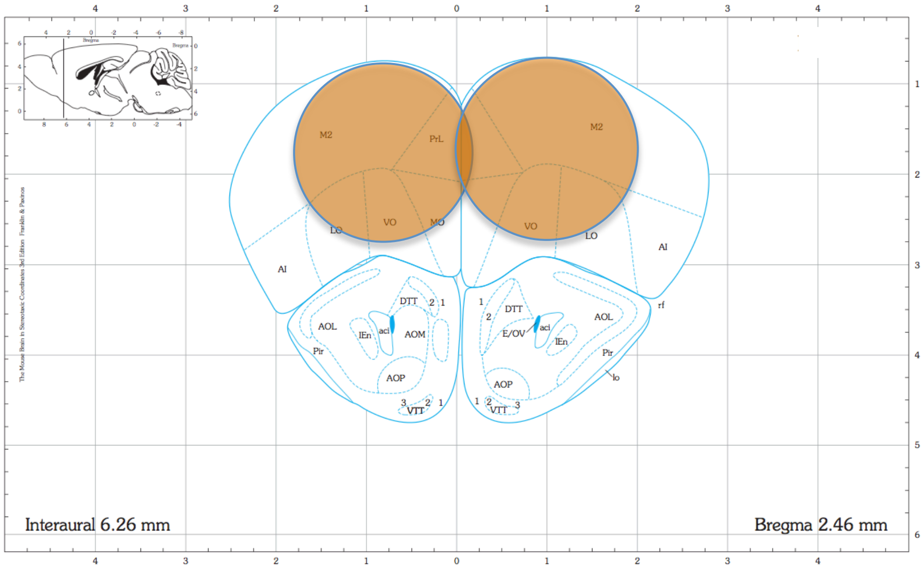

Bregma (+2.70)..(+2.40)

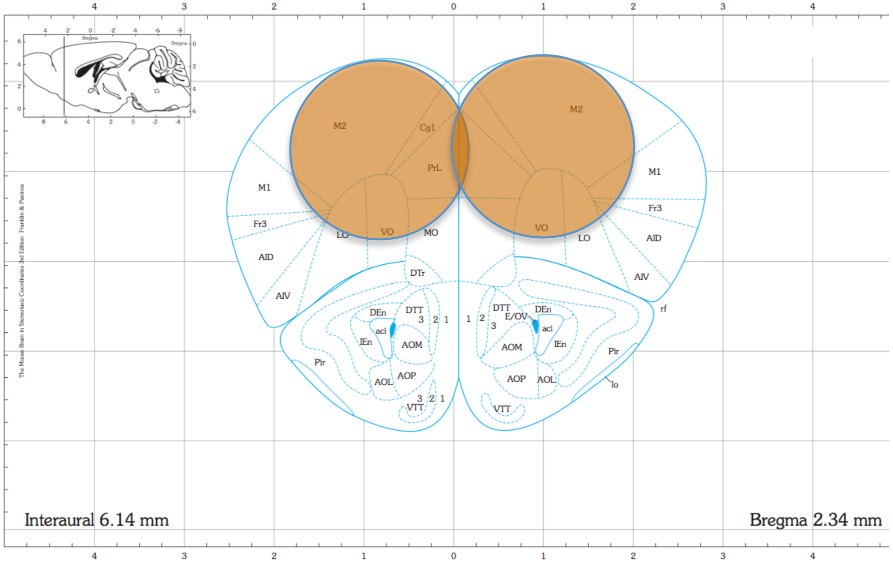

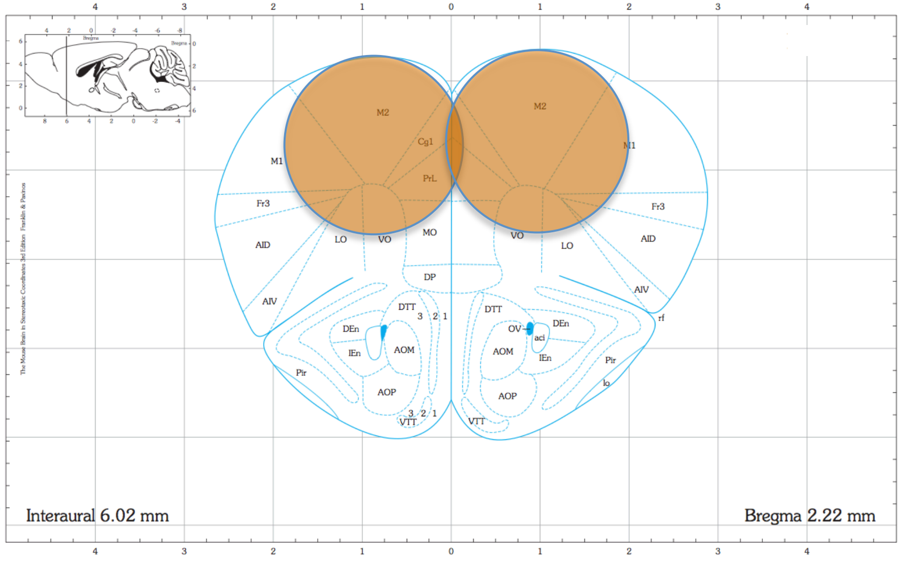

Plate Full Screen On/Off Home <= =>

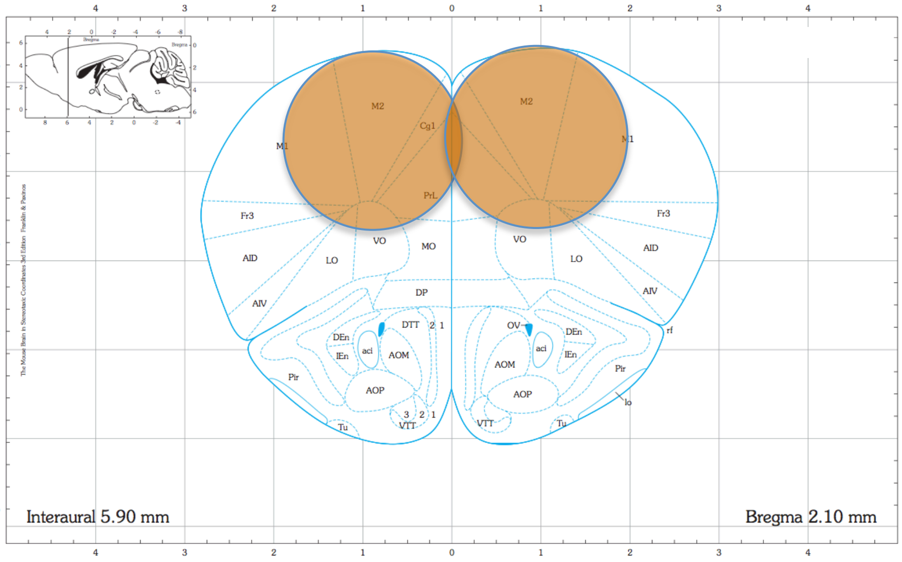

Bregma (+2.40)..(+2.10)

Click top left diagram to navigate to other levels. Point to an abbreviation to reveal full name.

Plate Full Screen On/Off Home <= =>

Micropunch Coordinates: NAC

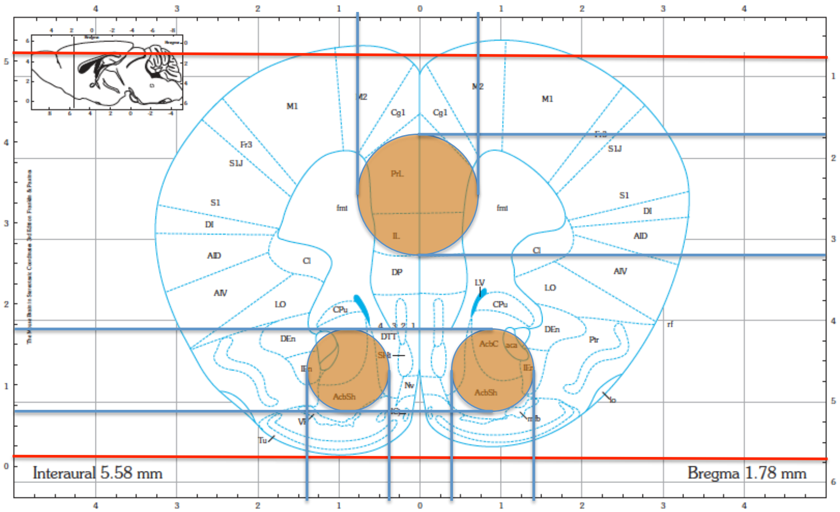

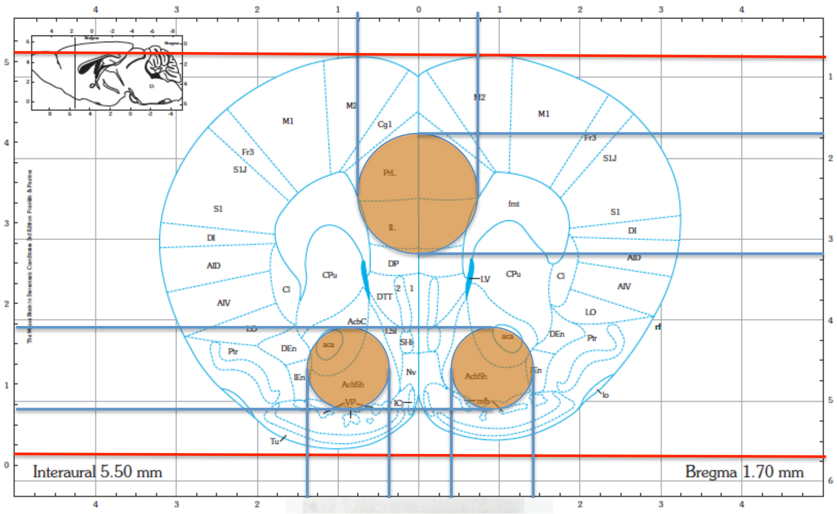

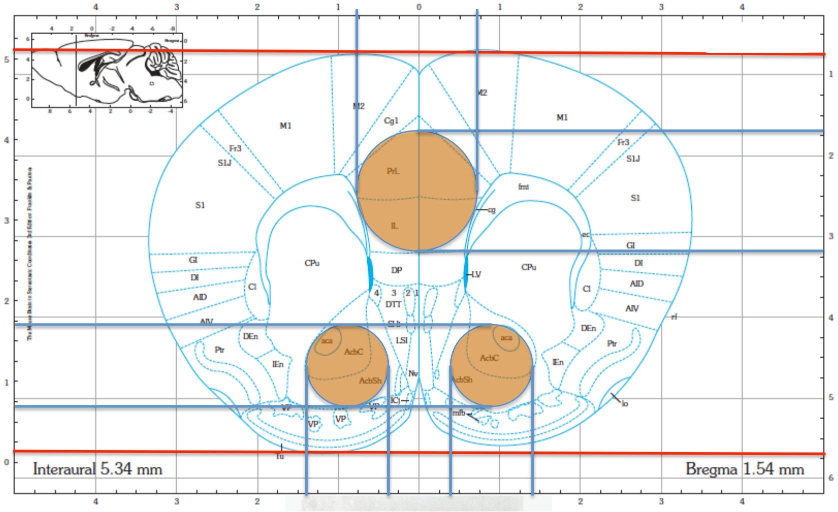

Bregma (+1.80)..(+1.20)

Micropunch Coordinates: AMY

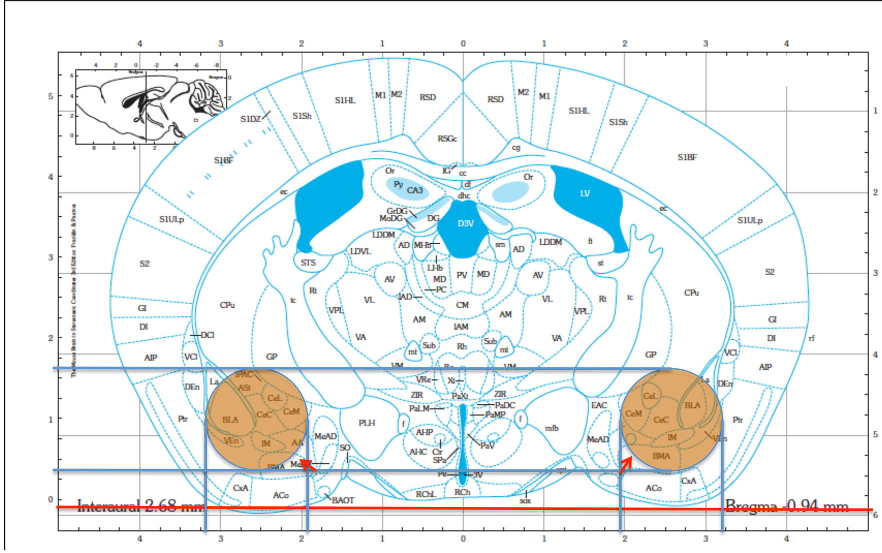

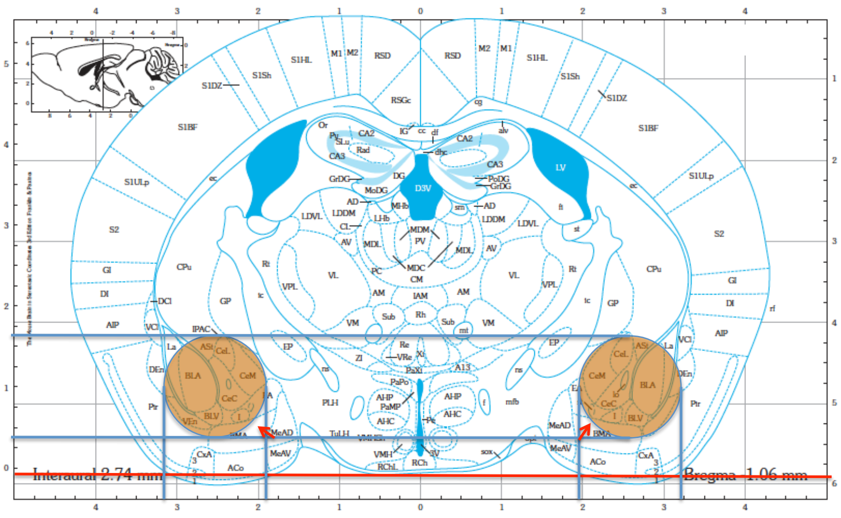

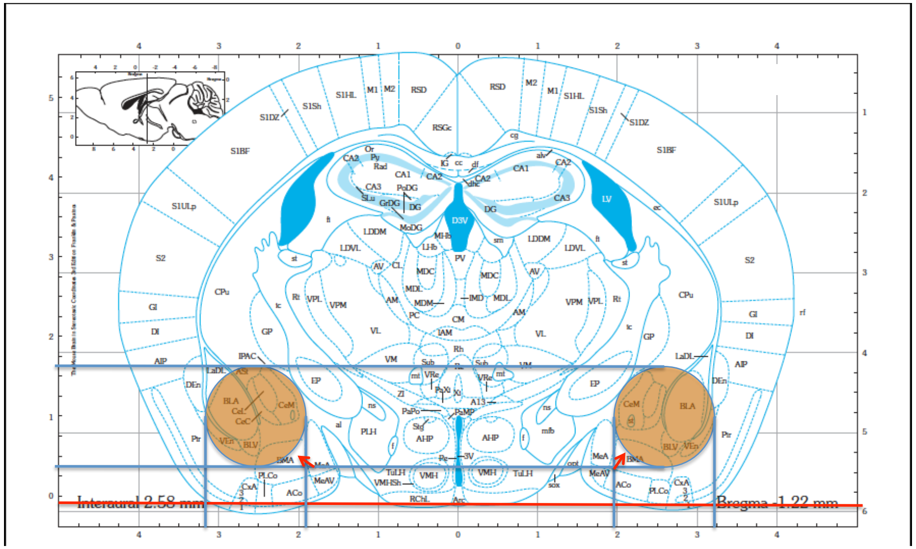

Bregma (-1.60)..(-0.90)
