# Supplementary figures and images for: Chronic Ethanol Exposure Produces Time- and Brain Region-Dependent Changes in Gene Coexpression Networks
Source: PLoS One. 2015 Mar 24;10(3):e0121522. doi: 10.1371/journal.pone.0121522 (PMC4372440; doi:10.1371/journal.pone.0121522)

**Cluster 1**

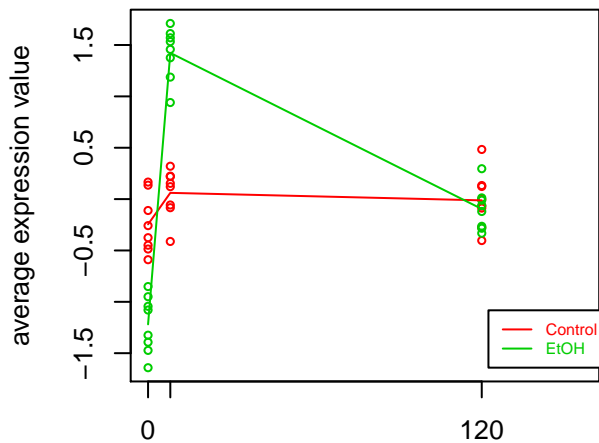

**Cluster 2**

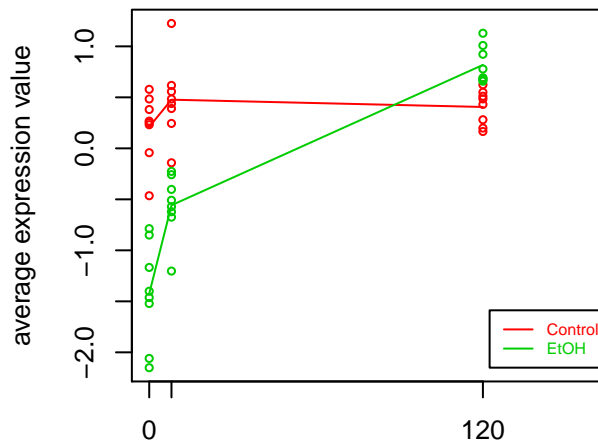

**Cluster 3**

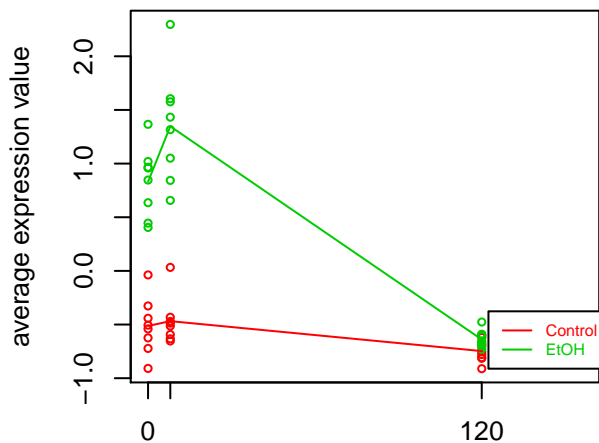

**Cluster 4**

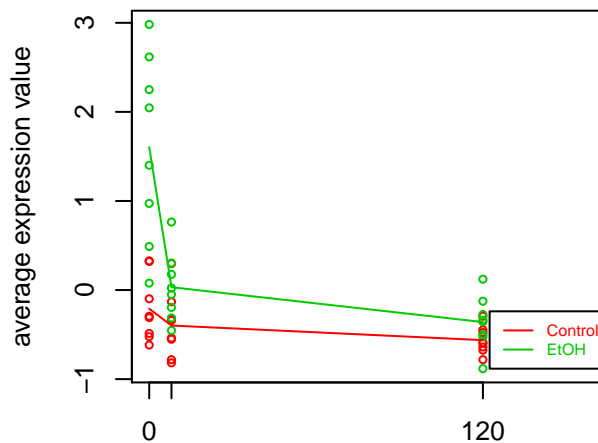

Supplement: S3 Fig — A two-step regression approach was used to identify clusters of differentially expressed genes with similar expression patterns across time. Each plot shows the hierarchical clustering (clusters = 4) of average expression profiles by time in the nucleus accumbens (NAC). The dots represent average expression values for each gene in the time series. (PDF) [file pone.0121522.s003.pdf]
