# Supplementary material for: Chronic Ethanol Exposure Produces Time- and Brain Region-Dependent Changes in Gene Coexpression Networks
Source: PLoS One. 2015 Mar 24;10(3):e0121522. doi: 10.1371/journal.pone.0121522 (PMC4372440; doi:10.1371/journal.pone.0121522)
Supplement: S4 Fig — A two-step regression approach was used to identify clusters of differentially expressed genes with similar expression patterns across time. Each plot shows the hierarchical clustering (clusters = 4) of average expression profiles by time in the prefrontal cortex (PFC). The dots represent average expression values for each gene in the time series. (PDF) [file pone.0121522.s004.pdf]

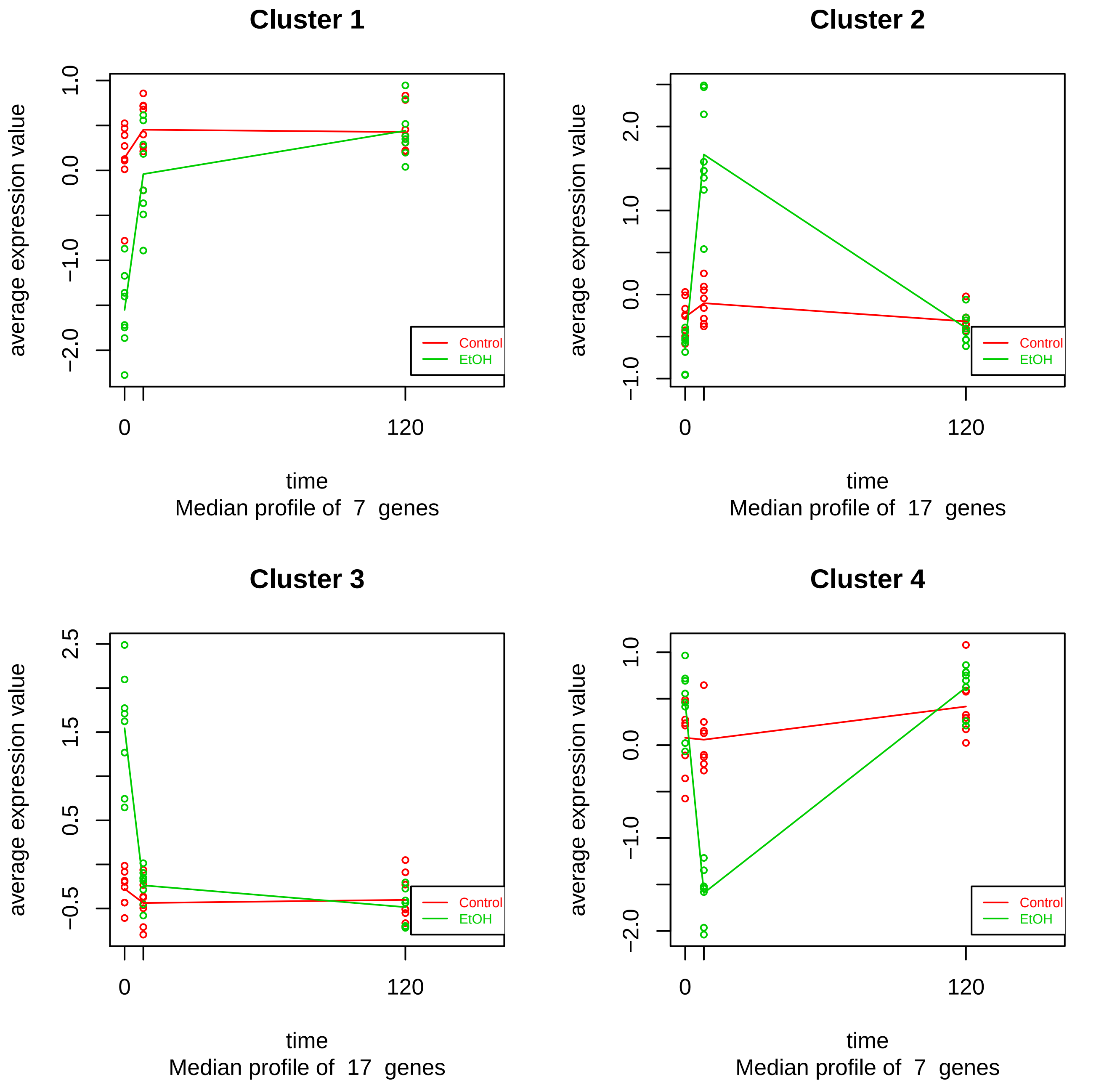
Cluster 1
average expression value
1.0
0.0
−1.0
−2.0
0
120
Control
EtOH
time
Median profile of 7 genes
Cluster 2
average expression value
2.0
1.0
0.0
−1.0
0
120
Control
EtOH
time
Median profile of 17 genes
Cluster 3
average expression value
2.5
1.5
0.5
−0.5
0
120
Control
EtOH
time
Median profile of 17 genes
Cluster 4
average expression value
1.0
0.0
−1.0
−2.0
0
120
Control
EtOH
time
Median profile of 7 genes
