# Supplementary material for: Chronic Ethanol Exposure Produces Time- and Brain Region-Dependent Changes in Gene Coexpression Networks
Source: PLoS One. 2015 Mar 24;10(3):e0121522. doi: 10.1371/journal.pone.0121522 (PMC4372440; doi:10.1371/journal.pone.0121522)
Supplement: S5 Fig — A two-step regression approach was used to identify clusters of differentially expressed genes with similar expression patterns across time. Each plot shows the hierarchical clustering (clusters = 4) of average expression profiles by time in the Liver. The dots represent average expression values for each gene in the time series. (PDF) [file pone.0121522.s005.pdf]

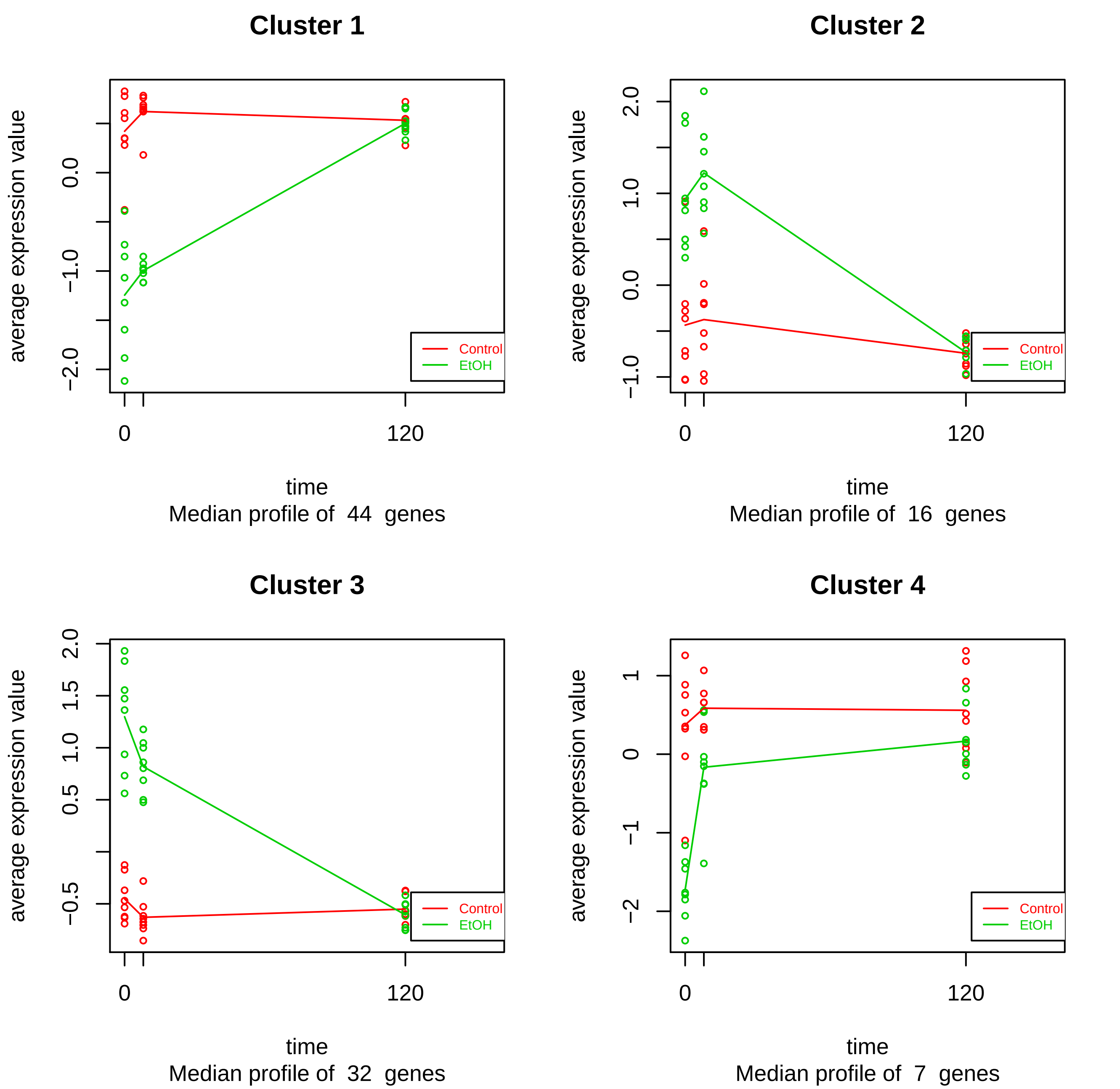

Cluster 1
average expression value
−2.0
−1.0
0.0
Control
EtOH
0
120
time
Median profile of 44 genes
Cluster 2
average expression value
−1.0
0.0
1.0
2.0
Control
EtOH
0
120
time
Median profile of 16 genes
Cluster 3
average expression value
−0.5
0.5
1.0
1.5
2.0
Control
EtOH
0
120
time
Median profile of 32 genes
Cluster 4
average expression value
−2
−1
0
1
Control
EtOH
0
120
time
Median profile of 7 genes
